# Impact of Cryopreservation Extenders on Epigenetic Changes in Bull Sperm: *H19* and *MEG3* Methylation

**DOI:** 10.1002/vms3.70688

**Published:** 2025-11-17

**Authors:** Razieh Fouladvandi, Ali Akbar Masoudi, Mohsen Sharafi

**Affiliations:** ^1^ Department of Animal Science Faculty of Agriculture Tarbiat Modares University Tehran Iran; ^2^ Department of Embryology at Reproductive Biomedicine Research Center Royan Institute for Reproductive Biomedicine ACECR Tehran Iran

**Keywords:** bull sperm quality, cryopreservation, DNA methylation, egg yolk extender, soybean lecithin extender

## Abstract

Cryopreservation of sperm is critical for livestock genetic improvement, yet its impact on epigenetic stability, especially the methylation of imprinted genes, remains unclear. This study aimed to compare the effects of soy lecithin (SLE) and egg yolk (EYE) extenders on sperm quality and the DNA methylation of the imprinted genes *H19* and *MEG3* following the cryopreservation process. We evaluated sperm motility parameters, membrane integrity and morphology using six Holstein‐Friesian bull sperm samples. Total motility (TM) showed significant reductions after cryopreservation, decreasing from 91.6% ± 1.52% in fresh sperm to 79.9% ± 1.52% in the SLE group and 77.3% ± 1.52% in the EYE group post‐thaw (*p* ≤ 0.05). Similarly, progressive motility (PM) decreased from 64.4% ± 1.8% in fresh sperm to 44.9% ± 1.8% in the (SLE) group and 39.6% ± 1.8% in the (EYE) group. Freezing and post‐thawing processes resulted in significant reductions (*p* ≤ 0.05) in other motility parameters, including linearity (LIN), curvilinear velocity (VCL), straight‐line velocity (VSL) and average path velocity (VAP). Following the freezing–thawing process, the SLE group exhibited a smaller reduction in PM and LIN compared to the EYE group. Notably, the SLE extender demonstrated a protective role in membrane integrity compared to the EYE extender (*p* ≤ 0.05). However, bisulphite sequencing revealed no significant differences in the methylation levels of *H19* and *MEG3* genes or in sperm morphology between the two extenders (*p* ≥ 0.05). This study highlights the importance of selecting appropriate extenders in cryopreservation protocols and their implications for future research on sperm quality and fertility.

## Graphical abstract

This study compared the cryoprotective effects of soy lecithin (SLE) and egg yolk (EYE) extenders on post‐thaw bull sperm quality and the methylation status of imprinted genes *H19* and *MEG3*.

The visual abstract outlines the experimental workflow: Bull sperm collection, dilution with either SLE or EYE extenders, followed by cryopreservation (symbolized by ice crystals and liquid nitrogen) and thawing (represented by a warming water bath).

Key results are presented visually and categorically:

Sperm quality analysis: Microscope‐based evaluation revealed that SLE was superior to EYE in preserving total motility, progressive motility and membrane integrity, as indicated by upward and downward directional arrows.

Epigenetic analysis: Bisulphite sequencing showed no significant differences in the DNA methylation patterns of the *H19* and *MEG3* genes between the two extenders, depicted by identical DNA helices.

The conclusion demonstrates that the soy lecithin‐based extender offers enhanced protection for sperm quality parameters without inducing significant epigenetic alterations in key imprinted genes.

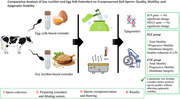

## 1 Introduction

In modern cattle industry, artificial insemination (AI) is the most widely used in worldwide. This technology is an effective tool for genetic improvement and therefore can contribute to the advancement of animal production (Faraji‐Arough et al. 2019; Nazari et al. 2021). Cryopreservation is a critical part of this process that includes diluting and cooling the semen samples, exposed on liquid nitrogen vapour and storage in liquid nitrogen (Ugur et al. 2019). Despite substantial advancements in cryoprotectant formulations and freezing protocols, post‐thaw sperm survival remains suboptimal, limiting the full potential of AI programs (Berean et al. 2024). Beyond conventional cellular damage, cryopreservation has been increasingly recognized as an epigenetic stressor capable of altering the sperm methylome and chromatin structure (Paoli et al. 2019).

The formation of intracellular and extracellular ice crystals during freezing damages sperm by causing membrane fragility through mechanical damage, disrupting osmotic balance and subsequently exposing the sperm genome to harmful changes (Ramazani et al. 2023). Furthermore, the functional and structural integrity of sperm is compromised during the freezing process due to oxidative stress, which results from elevated reactive oxygen species (ROS) production or impaired antioxidant defences. This imbalance between ROS production and the sperm's intrinsic antioxidant system leads to molecular and cellular damage, contributing to reduced motility, viability and fertility. Such oxidative disturbances may also disrupt DNA–protein interactions and histone retention patterns, which are closely linked to the epigenetic programming of spermatozoa (Moazamian et al. 2025). Importantly, emerging evidence suggests that oxidative events during the freeze–thaw process can also induce transcriptomic and epigenetic modifications, potentially diminishing reproductive outcomes (Fouladvandi et al. 2024; Salehi et al. 2020).

Epigenetic alterations, particularly changes in DNA methylation, have been associated with sperm quality, embryonic development and offspring health (Olszewska et al. 2022). For example, cryopreservation has been shown to increase DNA methylation levels in equine sperm, correlating with reduced conception rates (Aurich et al. 2016), whereas in avian species, methylation levels may decrease significantly after thawing (Salehi et al. 2020). These contrasting results highlight species‐specific epigenetic responses to cryopreservation and emphasize the importance of characterizing bovine sperm methylation dynamics. In bulls, differences in sperm methylome patterns have been linked to fertility status, with hypomethylation observed in low‐fertility sires (Štiavnická et al. 2022). However, whether cryopreservation induces targeted modifications at specific imprinted loci or results in broader methylation shifts remains poorly understood and represents a critical gap in bovine reproductive research.

Epigenetic regulation is essential for maintaining sperm functionality and ensuring proper embryonic development. Within the broad spectrum of epigenetically regulated loci, imprinted genes are particularly susceptible to environmental and procedural perturbations such as cryopreservation. Among these, H19 and *MEG3* have been widely investigated due to their critical roles in genomic imprinting, chromatin dynamics and germline epigenetic programming (Kläver et al. 2012). H19 encodes a maternally expressed lncRNA involved in testicular development and spermatogenesis, and aberrant methylation of its DMR has been correlated with impaired sperm motility, reduced quality and subfertility. *MEG3*, another imprinted lncRNA, contributes to chromatin integrity and genomic stability through interactions with p53‐mediated pathways and has been associated with variations in sperm quality and fertilization potential in livestock species. Disruptions in the methylation status of these loci may reduce fertilization efficiency and compromise reproductive performance in cattle breeding programs (Cannarella et al. 2023; Hosseini et al. 2024). Focusing on these genes enables a more mechanistic understanding of cryopreservation‐induced epigenetic changes and their implications for animal fertility management.

On the other hand, the development of cryoprotectants to reduce oxidative events is a common practice, but it seems that the necessary studies on the effect of cryoprotectants on epigenetic parameters have not been done. Moreover, extenders optimized solely for motility and viability may not necessarily ensure epigenetic stability, which could have downstream implications for embryonic competence and transgenerational health. This study aimed to assess the effects of cryopreservation on sperm cellular quality and to compare the protective effects of effects of soy lecithin (SLE) and egg yolk (EYE) in preserving *H19* and *MEG3* methylation, as well as post‐thaw quality parameters. By integrating classical semen evaluation with locus‐specific methylation analysis, this work contributes to a more mechanistic understanding of cryopreservation‐induced epigenetic perturbations in bovine sperm.

## 2 Materials and Methods

### 2.1 Chemicals, Reagents and Semen Collection

All chemicals and media components used in this study were primarily sourced from Sigma‐Aldrich (St. Louis, MO, USA) and Merck (Darmstadt, Germany), unless noted otherwise. Semen samples were collected twice weekly from six genetically verified Holstein Friesian bulls using an artificial vagina over a period of three consecutive weeks, resulting in a total of 36 ejaculates. Before processing, semen quality was thoroughly assessed for volume, colour, absence of urine and blood contamination, concentration (minimum 1 × 10^9^ sperm/mL), motility (at least 70%) and morphology (maximum of 10% abnormal forms). To minimize individual variability, the samples were pooled and divided into three equal portions. These aliquots were then allocated to the following experimental groups: (1) fresh semen, (2) semen frozen with a 1% soybean lecithin and (3) semen frozen with a 20% EYE extender. This approach ensured that the comparative effects of the extenders on sperm quality could be accurately assessed. The selection of 1% soybean lecithin was based on a substantial body of interspecies evidence suggesting that this concentration provides a favourable balance between cryoprotective efficacy and the avoidance of undesirable effects such as increased viscosity or potential cytotoxicity at higher doses. Although some studies have reported beneficial outcomes at concentrations above 1%, several investigations in bovine ovine and caprine models have shown that 1% lecithin is often sufficient to preserve sperm quality after thawing without compromising sample performance (Forouzanfar et al. 2010; Masoudi et al. 2016; Sun et al. 2021). Accordingly, this concentration was considered appropriate for assessing both conventional semen parameters and potential epigenetic alterations in the current study.

### 2.2 Preparation of Extenders and Cryopreservation Process

The Tris‐based medium was prepared following the procedure outlined by Moussa et al. (2002) and modified according to recent protocols (Sun et al. 2020). A Tris‐citric acid buffer was prepared by dissolving 2.4 g of TRIS (hydroxymethyl‐aminomethane), 1 g of citric acid, 1 g of fructose and 6.4 mL of glycerol in 100 mL of distilled water. To this solution, 25 mg of gentamicin, 50,000 IU of penicillin and 300 µg/mL of streptomycin were added and the mixture was filtered (0.22 µm) to ensure sterility. The pH of the extender was adjusted to 6.6 and then stored at −20°C until use. All solutions were prepared fresh weekly and protected from light to avoid component degradation. To ensure accurate evaluation of sperm motility, morphology and concentration in fresh semen, all samples were diluted 1:10 with the same Tris‐citric acid buffer used in the extenders, but without any cryoprotectants or supplements. This dilution reduced viscosity and sperm concentration, allowing reliable microscopic evaluation and CASA‐based analysis, while avoiding any confounding effects from extender components. For the preparation of the EYE and soybean lecithin‐based extenders, 1 g of soybean lecithin or 20% (v/v) fresh hen EYE was added to 100 mL of the prepared Tris buffer.

After evaluating the semen quality, aliquots of the ejaculate were diluted at room temperature with the prepared SLE and EYE extenders. The diluted semen samples were then aspirated into 0.25 mL French straws (IMV, L'Aigle, France) to achieve a concentration of 100 × 10^6^ spermatozoa/mL. These straws were sealed with polyvinyl alcohol powder and equilibrated at 4°C for 90 min. Following equilibration, the straws were frozen by placing them 5 cm above liquid nitrogen (LN_2_) vapour for 10 min, after which they were plunged into the LN_2_ (−196°C) for storage. For evaluation, the frozen straws were thawed at 37°C for 30 s in a water bath (immediately before analysis to maintain uniformity across sample).

### 2.3 Evaluation of Cellular Parameters of Sperm Post‐Thawing

#### 2.3.1 Motion Characteristics

Sperm motility was assessed using a computer‐assisted sperm analyser (SCA v6, Microptic SL, Barcelona, Spain) following established protocols (Shah et al. 2016), with modifications specific to bovine samples (Belala et al. 2024). Fresh and frozen–thawed semen aliquots (6 µL) were loaded into pre‐warmed (37°C) Leja counting chambers (20 µm depth; Leja Products, the Netherlands) and examined under phase‐contrast microscopy (Olympus Corporation, Tokyo, Japan) using a 10× objective (contrast setting: 169; brightness: 470). Image sequences were captured at 50 frames per second, with 30 consecutive frames analysed per sperm track. CASA settings were defined as follows: static sperm (average path velocity [VAP] < 7 µm/s), motile sperm (VAP ≥ 7 µm/s) and progressively motile sperm (VAP ≥ 25 µm/s, STR > 70%, linearity [LIN] > 50%), where STR = straight‐line velocity [VSL]/VAP × 100 and LIN = VSL/curvilinear velocity [VCL] × 100. Strict particle size gating (5–190 µm^2^) and a minimum track point threshold (≥18 consecutive points) were applied to ensure accurate sperm identification (Canonico et al. 2024). Additional kinematic parameters recorded included VCL, VSL, VAP, amplitude of lateral head displacement (ALH) and beat‐cross frequency (BCF). Quality control procedures included daily calibration using 5 µm certified latex beads (Microptic QC Kit), confirmation of chamber temperature stability (±0.5°C) and analysis by a single experienced operator to ensure consistency, with inter‐assay variation maintained below 5% (CV <5%) during validation trials.

#### 2.3.2 Functional Integrity of Membrane

**FIGURE 1:**
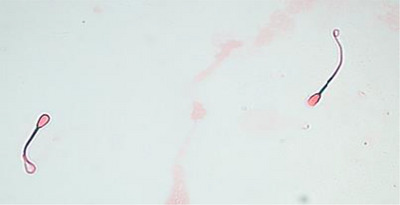
Evaluation of Sperm membrane integrity using the hypo‐osmotic swelling (HOS) test. Spermatozoa with swollen tails were quantified as the percentage of those exhibiting high membrane integrity (HOS‐positive), Magnification: ×400.

In this study, the integrity of bovine sperm plasma membranes was evaluated using the hypo‐osmotic swelling (HOS) test. A hypo‐osmotic solution with an osmolarity of 150 mOsm/kg, containing fructose (9 g/L) and sodium citrate (4.9 g/L), was prepared according to the optimized protocol for bovine sperm (Elkhawagah et al. 2024; Liaudat et al. 2023). Then, 10 µL of semen was mixed with 100 µL of the hypo‐osmotic solution (1:10 ratio) and incubated at 38°C for 30 min. Following incubation, the samples were examined under a phase‐contrast microscope at 400× magnification, and a minimum of 200 spermatozoa were evaluated. Spermatozoa exhibiting swollen or coiled tails were considered HOS‐positive. Samples with >60% HOS‐positive spermatozoa were considered to have optimal membrane integrity. A representative micrograph illustrates spermatozoa with high membrane integrity, characterized by tail swelling in the HOS test (Figure 1). This image highlights typical HOS‐positive sperm, reflecting effective membrane functionality.

#### 2.3.3 Sperm Morphology Assessment Pre‐ and Post‐Freezing

Sperm morphology was assessed using Hancock's solution (Hancock 1956). The solution was prepared by mixing 62.5 mL of 37% formalin, 150 mL of sodium saline (9.01 g NaCl in 500 mL distilled water) and 150 mL of buffer (200 mL of Solution A: 21 g Na_2_HPO_4_·2H_2_O in 500 mL water; 80 mL of Solution B: 22.254 g KH_2_PO_4_ in 500 mL water) in 500 mL of distilled water. For evaluation, 30 µL of the solution was placed on a slide, covered with a coverslip and examined under a phase‐contrast inverted microscope at 400× magnification. A total of 200 sperm cells were counted, and the percentage of abnormal sperm (abnormal acrosomes, detached heads, tail defects and midpiece abnormalities) was calculated and reported.

### 2.4 Evaluation of Gene *H19* and *MEG3* Methylation by Bisulphite Cloning and Sequencing

#### 2.4.1 DNA Extraction

DNA extraction from frozen semen (SLE and EYE) was performed as follows: 400 µL of semen was washed 4–6 times with 500 µL of 3 M Tris–HCl, followed by centrifugation for 5 min at 3000 × *g*, and removal of the supernatant using a modified phenol–chloroform protocol adapted from Xavier et al. (2018), optimized for sperm‐specific applications. Then, 500 mL SE buffer (1‐M Tris–HCl pH8.0; 3‐M NaCl; 0.5‐M EDTA; 20% sodium dodecyl sulphate), 30 µL Triton‐X100 (2%), 30 µL DTT (dithiothreitol; 1 M) and 60 µL proteinase K (10 mg/mL) were added. The samples were well mixed and incubated at 72°C for 15 min. A short‐controlled sonication step (e.g., 2 min, 15‐s pulses) was included to facilitate sperm tail detachment. Then were added 50 µL NaCl 6 M and well vortexed. Thereafter adding one volume of buffer‐saturated chloroform (Invitrogen) was centrifuged for 15 min at 4000 × *g* at room temperature, and the supernatant was transferred into a new 1.5‐mL tube. Then, 1/10 of a volume, 3 M sodium acetate (pH = 5.2) and two volumes of 96% cold ethanol were added and gently mixed to precipitate DNA. DNA was collected and transferred into a new 1.5‐mL tube and after centrifugation at 11,000 × *g* at RT for 15 min. DNA pellets were washed with 70% ethanol, dried, dissolved in 100 mL of TE buffer and kept at −20°C before PCR reaction. This optimized protocol yielded ∼23% higher DNA recovery (15–25 µg/400 µL sample) with high purity (A260/A280 = 1.7–1.9), and electrophoretic analysis confirmed intact DNA suitable for bisulphite conversion.

#### 2.4.2 Primers Design for Bisulphite Sequencing

We designed specific primers for amplification and sequencing with MethPrimer (http://www.urogene.org/methprimer/) and the software of PerlPrimer v6.01 (http://perlprimer.sourceforge.net).

In the initial step, an in silico bisulphite conversion simulation was performed, wherein all non‐CpG cytosines were converted to thymine, whereas CpG sites remained unmodified. This simulation accurately reflects the changes induced by sodium bisulphite treatment. Subsequently, the modified sequences were aligned using the BLAST tool in the NCBI database (https://www.ncbi.nlm.nih.gov/) to confirm primer binding specificity.

To enhance the sensitivity and specificity of the assay, a Semi‐Nested PCR strategy was employed. In the first PCR step, outer primers (F1/R1) were used, generating amplicons of 607 bp for *H19* and 223 bp for *MEG3*. The second PCR step involved a combination of an inner primer (F2) and the outer reverse primer (R1), yielding shorter (324 bp for *H19*) and more specific products. This hierarchical approach improves detection sensitivity compared to conventional PCR. All PCR steps were performed using an Eppendorf Mastercycler Pro thermocycler (Germany) under optimized conditions. The Primers and AT (annealing temperature) were Listed in Table 1.

**TABLE 1:** The characteristics of the primers used.

Gene symbol		Primer sequence (5′–3′)	Annealing temperature (°C)	PCR product size (bp)
*H19*	Outside forward	ATGGGTATGAGAGATAGAATAGTATTT	57	659
*H19*	Inside forward	GTTTTTGGTTATTTTTGTTTATTTAGT	55	376
*H19*	Reverse	TCTCACCTTATCATCTTAAAAATTC	57	
*MEG3*	Outside forward	AAAATTAGATGGTAGGTGAGATTAGGTTT	56	279
*MEG3*	Inside forward	ATGGTAGGTGAGATTAGGTTTTT	55	270
*MEG3*	Reverse	ACTCACCCCAAACCAAACAACAA	56	

#### 2.4.3 PCR Amplification, Cloning and Sequencing

Genomic DNA from frozen sperm was bisulphite converted performed using the EpiTect Bisulphite Kit (Qiagen, USA) according to manufacturer instructions. Following bisulphite treatment, amplification and sequencing, convert unmethylated cytosines to thymine and methylated cytosine residues remain as cytosine (Daigneault et al. 2020). Prior to cloning, PCR products were purified using the QIAquick PCR Purification Kit to remove primer dimers and contaminants, ensuring high‐quality DNA for subsequent cloning steps. PCR products were cloned into *Escherichia coli* DH5α, TA cloning vectors (Invitrogen, Carlsbad, CA, USA) and sequenced. TA cloning was performed using the pTZ57R/T vector, which allows a ligation of Taq polymerase‐amplified PCR products due to complementary 3′ adenine overhangs. Ligation was conducted with T4 DNA Ligase at 16°C overnight. Next, 10 single white colonies (positive for the bisulphite treated insert) were selected and the cloned ones for further PCR amplification were amplified with M13 universal primers in AT of 54°C to confirm the correct insert size. Competent *E. coli* DH5α cells were prepared by calcium chloride treatment and transformed via heat shock. Transformed cells were plated on selective LB agar containing ampicillin, IPTG and X‐Gal for blue–white screening to identify recombinant colonies.

**FIGURE 2:**
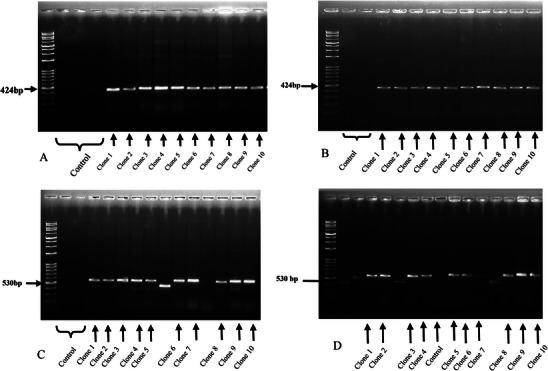
Representative gel electrophoresis of PCR products amplified from Bisulphite‐treated DNA of bull sperm for H19 and MEG3 genes in soy lecithin (SEL) and egg yolk extenders (EYE). Genomic DNA from frozen sperm was bisulphite‐treated and then amplified using M13 universal primers. PCR products were cloned into TA vectors, transformed into *Escherichia coli* DH5α and sequenced. The gel displays results from 10 selected white colonies positive for the bisulphite‐treated insert, with bands corresponding to the expected insert sizes of 424 bp for the *H19* gene and 530 bp for the *MEG3* gene. Panels (A) and (B) show *MEG3* gene amplification in egg yolk (ME) and soy lecithin (MS) extenders, respectively, whereas Panels (C) and (D) illustrate *H19* gene amplification in egg yolk (HE) and soy lecithin (HS) extenders.

The PCR product was analysed by BiQ Analyzer software (Max Planck Institute for Informatics). As shown in Figure 2, the gel electrophoresis results confirm the amplification of PCR products and the expected insert sizes. Colony PCR using universal M13 primers was performed to verify the presence and correct the insertion of the target sequence before sequencing.

### 2.5 Statistical Analysis

Each treatment was replicated six times. Prior to analysis, data normality was assessed using the Shapiro–Wilk test with the univariate procedure in SAS 9.1 (SAS Institute Inc., Cary, NC, USA). The results are expressed as the mean percentage ± standard error of the mean (SEM). However, for methylation data, which are categorical and proportional by nature, SEM was not reported. Instead, a cloning‐based method was used to determine the proportion of methylated CpG sites per clone, and Fisher's exact test was employed for group comparisons. Statistical comparisons between fresh and cryopreserved sperm were conducted using paired Tukey's test at a significance level of (*p* ≤ 0.05). The assumption of normality was satisfied for all variables, thereby justifying the use of parametric methods in the subsequent analyses. The statistical model applied in this study was as follows:

$$Y_{i}=\mu +T_{i}+R_{j}+e_{ij}$$

where *Y_i_* represents the observed dependent variables (including sperm parameters), *μ* is the population mean, *T_i_* denotes the treatment effect, *R_j_* accounts for the replication effect, and *e_ij_* represents the random residual error.

## 3 Results

### 3.1 Sperm Motility and Kinematic Characteristics Pre‐ and Post‐Freezing

Sperm motility and kinematic parameters were analysed using CASA for fresh and frozen sperm in soybean lecithin and EYE‐based extenders (Table 2). Fresh sperm showed significantly higher total motility (TM) (91.6% ± 1.52%) and progressive motility (PM) (64.4% ± 1.8%) compared to frozen sperm (*p* ≤ 0.05). Among the frozen groups, a significant difference (*p* ≤ 0.05) was observed in PM, with SLE showing higher values (44.9% ± 1.8%) than EYE (39.6% ± 1.8%). Fresh sperm also had the highest VAP (36.7 ± 2.5 µm/s) and VSL (29.7 ± 2.9 µm/s), with no significant differences among frozen treatments (*p* ≥ 0.05). Velocity of the sperm along the path (VCL) was highest in fresh sperm (65.9 ± 3.2 µm/s). No significant differences in STR (directness of movement) were found among treatments (*p* ≥ 0.05), but significant differences in LIN were observed (*p* ≤ 0.05), with fresh sperm showing the highest LIN (52.2% ± 1.8%) and EYE‐based frozen sperm the lowest (37.2% ± 1.8%).

**TABLE 2:** The effects of different extenders on the motion parameters of bull sperm in after freezing–thawing.

Spermatozoa	Fresh	SLE	EYE
Total motility	91.6 ± 1.52^a^	79.9 ± 1.52^b^	77.3 ± 1.52^b^
Progressive motility (%)	64.4 ± 1.8^a^	44.9 ± 1.8^b^	39.6 ± 1.8^c^
VAP (µm/s)	36.7 ± 2.5^a^	25 ± 2.5^b^	23.6 ± 2.5^b^
VSL (µm/s)	29.7 ± 2.9^a^	17.6 ± 2.9^b^	19.8 ± 2.9^b^
VCL (µm/s)	65.9 ± 3.2^a^	45.5 ± 3.2^b^	42.2 ± 3.2^b^
STR	55.9 ± 3.6	52.6±3.6	54.1±3.6
LIN (%)	52.2 ± 1.8^a^	45.1 ± 1.8^b^	37.2 ± 1.8^c^

*Note*: Data are expressed as mean ± SEM. Different letters within the same column show significant differences among the groups at (*p* < 0.05).

Abbreviations: EYE, egg yolk; LIN, linearity; PM, progressive motility; SEM, standard error of the mean; SLE, soybean lecithin; TM, total motility; VAP, average path velocity; VCL, curvilinear velocity; VSL, straight linear velocity.

### 3.2 Integrity of Membrane and Morphology of Bull Sperm Before and After Cryopreservation

**FIGURE 3:**
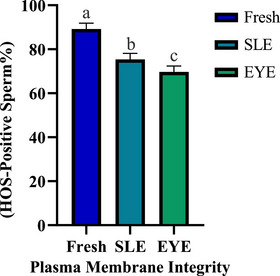
Plasma membrane integrity (% HOS‐positive sperm) in fresh and frozen–thawed sperm with SLE or EYE extenders (mean ± SEM). Different superscripts (a–c) indicate significant differences (*p* ≤ 0.05). EYE, egg yolk; HOS, hypo‐osmotic swelling; SLE, effects of soy lecithin.

**FIGURE 4:**
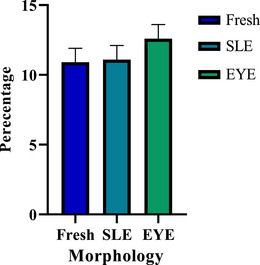
Comparison of abnormal sperm morphology in different groups (mean ± SEM). EYE, egg yolk; SLE, effects of soy lecithin.

Figure 3 shows the membrane integrity data, whereas Figure 4 illustrates the abnormal morphology results.

The analysis of abnormal sperm morphology revealed no significant differences among the three treatment groups (*p* ≥ 0.05), indicating that the freezing process did not impact bull sperm morphology. Furthermore, although no significant differences in membrane integrity were observed between the two cryopreserved groups (*p* ≥ 0.05), both exhibited a significant decrease compared to the fresh sperm group (*p* ≤ 0.05). The fresh sperm group demonstrated the highest percentage of sperm with intact membranes (89.2% ± 2.7%).

### 3.3 Evaluation of *H19* and *MEG3* Genes Methylation

#### 3.3.1 DNA Sequencing

**FIGURE 5:**
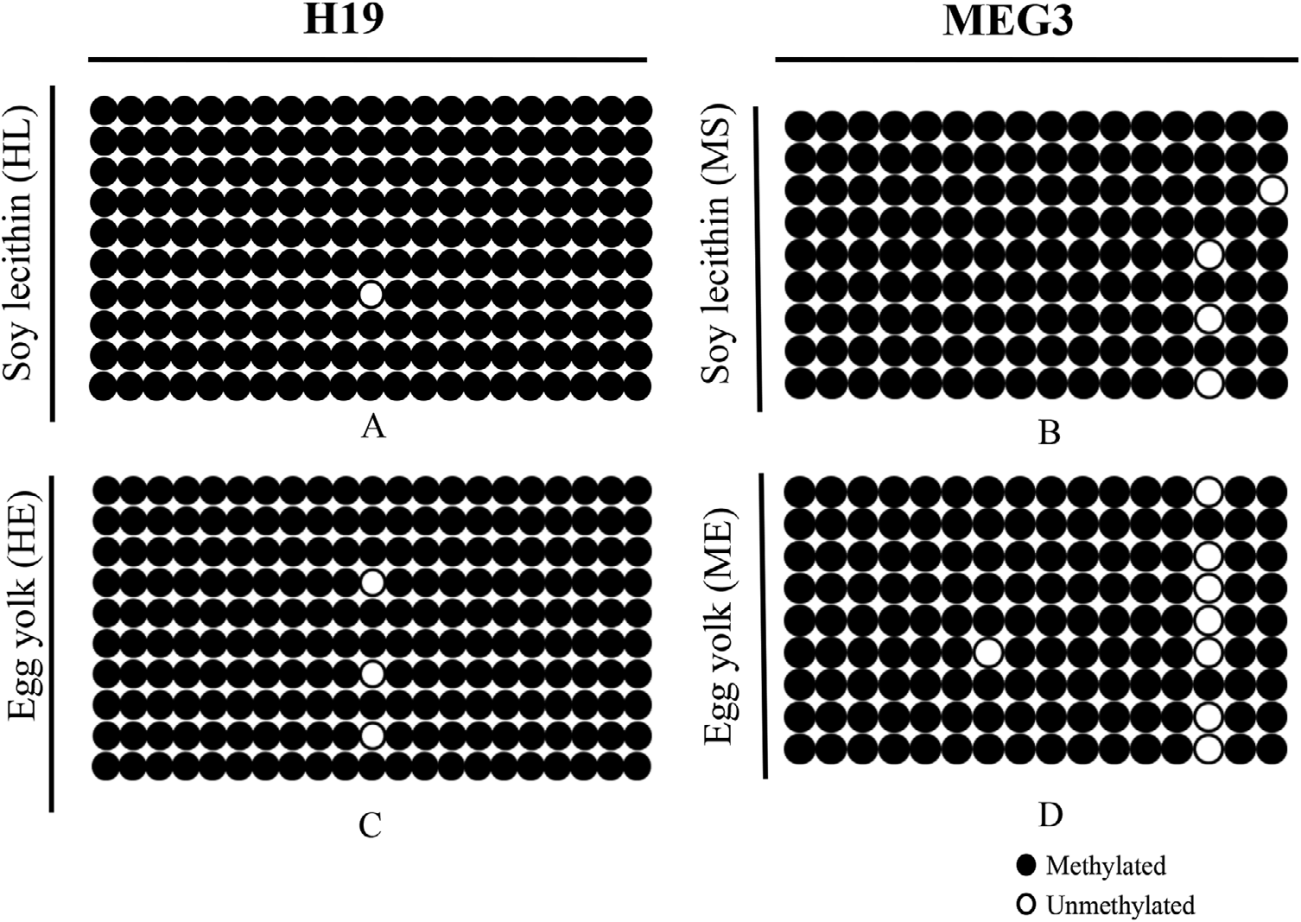
Bisulphite sequencing profiles of the *H19*, *MEG3* genes in frozen groups (soy lecithin and egg yolk). Scores for the methylation of each CpG were obtained by sequencing PCR clones derived from bisulphite‐treated genomic DNAs. The individual profiles of sequences of the imprinted *H19* and *MEG3* genes with every CpG dinucleotide represented by a circle. Evaluated 21 CpG for *H19* and 16 CpG for *MEG3* genes. Black circles represent the methylated CpGs site and open circles, unmethylated CpGs site. Individual line shows a bacterial clone, which was sequenced and each circle one single CpG dinucleotide in the regions analysed. (A) *H19* gene in soy lecithin (HS). (B) *MEG3* gene in soy lecithin (MS), (C) *H19* gene in egg yolk (HE) and (D) *MEG3* in egg yolk (ME).

DNA methylation pattern of *H19* and *MEG3* genes were analysed in bull post‐thawed sperm. For each sample, 10 clones from *H19*, *MEG3* PCR products were sequenced. The methylation status of all CpGs present in the sequences was analysed manually and using the BiQ Analyzer software (Figure 5).

**FIGURE 6:**
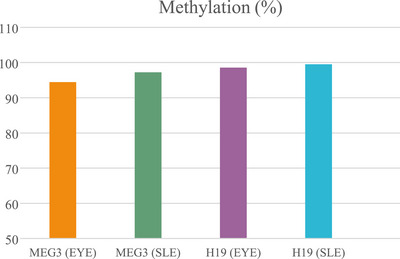
Comparison of DNA methylation in *H19* and *MEG3* genes under the two different extenders (SLE and EYE). No significant differences were found (*p* > 0.05). EYE, egg yolk; SLE, effects of soy lecithin.

The percentage of DNA methylation for *H19* gene in EYE was 98.57 (3/210). This value in SLE was 99.52% (1/210) of the CpGs methylated. For *MEG3* gene, clones with 94.44% (8/144) of the CpGs methylated in EYE and in SLE 97.22% (4/144) of the CpGs methylated (Figure 6). Despite a small different in DNA methylation, these differences were not significant between SLE and EYE in both of gene (*H19* and *MEG3*) (*p* ≥ 0.05).

## 4 Discussion

The cryopreservation of bull sperm is essential for genetic improvement programs and reproductive biotechnology. However, it induces a cascade of biophysical and biochemical stressors that adversely affect sperm motility, morphology and genomic stability, limiting its post‐thaw fertility potential (Arunkumar et al. 2022). Different cryoprotectants exhibit varying effects on sperm quality during cryopreservation across species due to their distinct biochemical properties and interactions with sperm cells (Mousavi et al. 2023).

In the present study, cryopreservation resulted in a marked reduction in several sperm motility parameters. Among these extenders, EYE formulations contain low‐density lipoproteins (LDLs), phospholipids, cholesterol and antioxidants that stabilize sperm membranes during cooling and freezing. LDLs form a protective layer that reduces mechanical and osmotic damage, whereas cholesterol and phosphatidylcholine help maintain membrane fluidity (Castro et al. 2025; Dalal et al. 2020). In contrast, SLE is composed mainly of plant‐derived phospholipids (e.g., phosphatidylcholine, phosphatidylethanolamine) and tocopherols, which integrate into the sperm membrane, enhance resistance to osmotic and oxidative stress and reduce lipid peroxidation, thereby preserving mitochondrial function (Hermansson et al. 2021).

Both extenders stabilize membrane lipids, prevent capacitation‐like changes and limit cryo‐induced apoptosis, but their relative success varies with formulation and species‐specific sperm membrane properties (Nguyen et al. 2019).

Meanwhile, commercial bull semen extenders based on soy lecithin and liposomes have demonstrated comparable post‐thaw sperm quality and fertility rates to EYE extenders (Lima‐Verde et al. 2018). These differences highlight the complex nature of cryoprotectant effectiveness and suggest that the optimal choice of soy lecithin‐based extender may depend on specific experimental conditions, formulations, concentrations and species‐related factors (Miguel‐Jimenez et al. 2020). For instance, in goats, SLE better preserved DNA and acrosome integrity while reducing lipid peroxidation compared to EYE (Chelucci et al. 2015). For ram sperm, SLE performed similarly to EYE in preserving motion characteristics and fertility rates (Masoudi et al. 2016). In canine semen, a 0.4% SL extender was more effective than EYE in maintaining sperm parameters during liquid storage (Kasimanickam et al. 2012).

As discussed in the manuscript, the literature reports inconsistent results regarding the efficacy of soy lecithin‐versus EYE‐based extenders (Khatun et al. 2021; Singh et al. 2018; Sun et al. 2020). Such inconsistencies likely reflect both extender‐specific effects and interspecies differences in sperm membrane architecture. For instance, bovine sperm possess higher cholesterol content, contributing to superior membrane stability during freezing (Upadhyay et al. 2021), whereas ovine sperm, with a lower ratio, are more cryo‐sensitive (Carro et al. 2020). Similarly, the seminal plasma profile—particularly the balance of enzymes and antioxidants—differs among species and may modulate sperm‐extender interactions. For example, cathepsin activity in boars can exacerbate membrane disruption, whereas elevated antioxidant capacity in stallions offers greater cryo‐protection (De Lazari et al. 2019).

Although it has been established that cryopreservation can cause significant morphological changes in bull sperm (Enciso et al. 2011; Gangwar et al. 2019), we did not observe any significant differences between the fresh sperm group and the cryopreserved groups. Research conducted by Behnam et al. (2023) found that although certain additives, such as Rho kinase inhibitors, can enhance specific aspects like motility and membrane integrity, the overall morphological structure of frozen–thawed sperm remains similar to that of fresh sperm.

In dogs, EYE extenders generally outperformed lecithin‐based ones, particularly in maintaining motility and membrane integrity (Axnér and Lagerson 2016). Dalmazzo et al. (2018) found that lower concentrations of soy lecithin performed similarly to EYE in preserving dog sperm motility and mitochondrial activity.

Although SLE shows promise in maintaining sperm motility and membrane integrity, its role in protecting against epigenetic alterations requires further investigation. Identifying the impact of sperm cryopreservation on epigenetic patterns, particularly in livestock like bulls, is critical for optimizing AI techniques in animal husbandry (Khan et al. 2021).

In this research, we investigated the effects of cryopreservation on the methylation of *H19* and *MEG3* genes, focusing on the efficacy of SLE and EYE in preserving sperm quality. Our findings highlight an important issue: Although the soybean lecithin‐based extender positively affects the functional parameters of bull sperm compared to the EYE extender during cryopreservation, it does not appear to induce significant changes in the methylation levels of the *H19* and *MEG3* genes. Despite observing slight differences in DNA methylation levels between the SLE and EYE extenders, these differences were not statistically significant (*p* ≥ 0.05). Other studies have revealed a complex and nuanced relationship between cryopreservation techniques and cryoprotectants, highlighting their impact on the stability of epigenetic patterns. A similar study was conducted on rooster sperm, comparing Lake and Beltsville extenders for cryopreservation. The Lake extender resulted in higher levels of H3K9 acetylation (17.4 ± 1.8) and H3K4 methylation (42 ± 2.3) compared to the Beltsville extender. However, no significant differences in DNA methylation were observed between the two extenders. Additionally, the Lake extender enhanced sperm motility, viability and fertility rates (59.5% vs. 47.2%), whereas the Beltsville extender was associated with higher levels of ROS and apoptosis. Overall, the Lake extender demonstrated greater effectiveness in improving sperm quality and epigenetic parameters (Salehi et al. 2020). In a similar study, our prior investigation into bovine sperm demonstrated that extenders based on soybean lecithin significantly outperformed EYE‐based extenders in preserving both histone acetylation and sperm quality in bulls (Fouladvandi et al. 2024). Research on sperm cryopreservation in aquaculture species, particularly *Colossoma macropomum* (Tambaqui), demonstrated that cryoprotectant agents (CPAs), such as dimethyl sulfoxide (DMSO), methanol and ethylene glycol, are effective for preserving sperm motility and fertilization capacity (de Mello et al. 2017).

In European eel, initial use of DMSO achieved satisfactory post‐thaw motility; nonetheless, concerns over epigenetic modifications prompted a shift to methanol as a preferred cryoprotectant (Herranz‐Jusdado et al. 2019). These studies underscore the need for careful selection of CPAs and the optimization of cryopreservation protocols to mitigate epigenetic alterations while preserving sperm viability for aquaculture and conservation. In the study conducted by Zeng et al. (2014), the effects of various freezing protocols on the expression of epigenetic‐associated genes in pigs were investigated. The study utilized three freezing conditions: 3% glycerol, a combination of 250 mM trehalose and 3% glycerol, and a combination of 1 mM glutathione and 3% glycerol. Additionally, three control groups were examined: direct freezing, programmed freezing with LEY and programmed freezing without LEY. The findings revealed that fresh sperm exhibited the highest expression levels of *Dnmt3a*, *Dnmt3b* and *Prm1* genes. In contrast, the programmed freezing treatments without LEY showed the lowest expression levels of *Dnmt3a* and *Prm1*. These results highlight the detrimental effects of freezing on the expression of epigenetic genes and suggest that the choice of cryoprotective agents can influence the extent of epigenetic alterations in sperm. Nevertheless, Lu et al. (2018) and Kläver et al. (2012) observed no significant changes in the methylation patterns of various imprinted genes, including *H19* and *MEST*, following cryopreservation. In contrast, Khosronezhad et al. (2023) found that cryopreservation led to increased methylation of the *PAX8*, *PEG3* and *RTL1* genes in human sperm, with vitrification causing more substantial changes than rapid freezing. Although we acknowledge this gap, the primary aim of our research was not centred on examining the effects of freezing on epigenetic markers. Nonetheless, assessing the initial methylation status of these genes could provide a more thorough understanding of the changes brought about by the freezing process. Future studies should aim to include fresh sperm data to address this gap and enhance the interpretation of how cryopreservation affects sperm epigenetics. Despite these limitations, our study contributes significant insights into the effects of cryopreservation and extender formulations on sperm motility and overall quality.

## 5 Conclusion

This study demonstrated that soybean lecithin–based extenders (SLE) more effectively preserve post‐thaw sperm motility and membrane integrity than EYE‐based extenders (EYE). Although no significant differences were detected in the methylation levels of *H19* and *MEG3*, this outcome may reflect locus‐specific epigenetic responses to cryopreservation stress, rather than indicating an overall lack of biological relevance. It is possible that other epigenetic loci or regulatory mechanisms are more sensitive to the compositional differences between extenders.

## Author Contributions

**Razieh Fouladvandi** was responsible for designing the study, collecting and analysing data, conducting statistical evaluations and drafting the manuscript. **Ali Akbar Masoudi** and **Mohsen Sharafi** contributed to manuscript preparation and provided significant critical revisions.

## Funding

The authors have nothing to report.

## Ethics Statement

The authors confirm that the ethical policies of the journal, as outlined in the author guidelines, have been adhered to, and approval from the appropriate ethics review committee has been obtained. Ethical approval for this research was granted by the Research Ethics Committees of and Royan Institute under the reference number IR.ACECR.ROYAN.REC.1396.168.

## Conflicts of Interest

The authors declare no conflicts of interest.

## Peer Review

The peer review history for this article is available at https://doi.org/10.1002/vms3.70688.</p>

## Sanctions Regulations and Laws

The authors are employed by the academic institution ‘Tarbiat Modares University’ where research or teaching is the primary function of the unit.

## Data Availability

The data that support the findings of this study are available on request from the corresponding author. The data are not publicly available due to privacy or ethical restrictions.
